# Immunity to the Dual Threat of Silica Exposure and *Mycobacterium tuberculosis*

**DOI:** 10.3389/fimmu.2018.03069

**Published:** 2019-01-09

**Authors:** Petr Konečný, Rodney Ehrlich, Mary Gulumian, Muazzam Jacobs

**Affiliations:** ^1^Centre for Environmental and Occupational Health, School of Public Health and Family Medicine, University of Cape Town, Cape Town, South Africa; ^2^Division of Immunology, Department of Pathology and Institute of Infectious Disease and Molecular Medicine, Faculty of Health Sciences, University of Cape Town, Cape Town, South Africa; ^3^National Health Laboratory Service, Department of Toxicology and Biochemistry, National Institute for Occupational Health, Johannesburg, South Africa; ^4^Division of Molecular Medicine and Haematology, University of the Witwatersrand, Johannesburg, South Africa; ^5^National Health Laboratory Service, Johannesburg, South Africa; ^6^Immunology of Infectious Disease Research Unit, South African Medical Research Council, Cape Town, South Africa

**Keywords:** tuberculosis, silica, immunity, macrophages, granulomas, T cell

## Abstract

Exposure to silica and the consequent development of silicosis are well-known health problems in countries with mining and other dust producing industries. Apart from its direct fibrotic effect on lung tissue, chronic and immunomodulatory character of silica causes susceptibility to tuberculosis (TB) leading to a significantly higher TB incidence in silica-exposed populations. The presence of silica particles in the lung and silicosis may facilitate initiation of tuberculous infection and progression to active TB, and exacerbate the course and outcome of TB, including prognosis and survival. However, the exact mechanisms of the involvement of silica in the pathological processes during mycobacterial infection are not yet fully understood. In this review, we focus on the host's immunological response to both silica and *Mycobacterium tuberculosis*, on agents of innate and adaptive immunity, and particularly on silica-induced immunological modifications in co-exposure that influence disease pathogenesis. We review what is known about the impact of silica and *Mycobacterium tuberculosis* or their co-exposure on the host's immune system, especially an impact that goes beyond an exclusive focus on macrophages as the first line of the defense. In both silicosis and TB, acquired immunity plays a major role in the restriction and/or elimination of pathogenic agents. Further research is needed to determine the effects of silica in adaptive immunity and in the pathogenesis of TB.

## Introduction

It has long been hypothesized that inhaled silica dust contributes to TB development and progression via its physicochemical and biological properties (1–3). However, while recent research has explored the role of silica in immune response impairment, the mechanisms of reaction to the combination of silica particles and *Mycobacterium tuberculosis* (Mtb) are poorly understood (4). In this review, the immunological responses to silica exposure and to Mtb are reviewed, either as independent pathways, or where evidence is available, in the context of co-exposure and dual disease.

### Silica and Silicosis

There are a number of pathologies associated with exposure to silica, including silicosis, lung malignancy, autoimmune disease and pulmonary infection, notably tuberculosis (5, 6).

Silicosis is a disease of the lower respiratory system caused by high dose and/or long-term inhalation of silica particles in mining and occupations involved in rock or sand processing, manufacturing of stone based products and utilization of sand as an abrasive agent (7–9). Silicosis results from the deposition of respirable silica particles in the lower respiratory tract, causing inflammation, collagen deposition, and fibrotic lesions. The outcome may vary from subclinical pathological changes to severe damage of lung tissue, diminished quality of life, and decreased lifespan (10). The development of a specific type of fibrotic granuloma the signal pathological lesion of silicosis is not restricted to the lungs but has also been observed in the liver, spleen, and bone marrow (11).

The prevalence of acute and severe silicosis due to very high exposures has decreased in the modern era and silicosis in general is uncommon in developed countries. However, silicosis remains prevalent in countries with extensive mining industries, such as China (6, 12, 13), South Africa (14, 15), Mexico (16), Brazil (17), and India (18), and continues to be recorded in the USA (19) and Australia (20). However, many countries do not register occupational diseases accurately and the enduring “residence time” of silica in the lungs after exposure, with a long asymptomatic phase, prevents early diagnosis (21).

The immune system response to silica particles has been extensively studied. Research has focused mainly on the initial interaction with cells residing in the lungs and the immunological modification of innate immune cellular responses. Special attention has been paid to the first encounters of silica with alveolar macrophages (AMs). In general, silica particles enter the alveolar space after inhalation and interact with macrophages, resulting in the engulfment of inhaled particles into the phagosome. The MARCO scavenger receptor has been described as the main molecule responsible for silica recognition and uptake (22), although other receptors from the group of Pattern Recognition receptors (PRRs) can be involved. It has been shown that CD204 may also interact with silica and inhibit the activity of macrophages. Some of the PRRs, such as Toll-like receptors (TLRs), are crucial in the response to bacterial infection. Significant downregulation of Toll-like receptor 2 (TLR2) following silica exposure might be one of the reasons contributing to higher TB susceptibility (23). Finally, the inability of macrophages to digest and eliminate phagocytized particles leads to persistent inflammation and modification of cellular responses (24).

The toxicity and pathogenicity profile of silica and thus the risk of silicosis vary with the size, physical and chemical properties of the inhaled particles (1, 25). A number of studies have focused on identification and characterization of specific toxic parameters (25–33). For example, different admixtures in dust can alter the biological activity of the silica particles (1, 34–38). While crystalline structure has long been accepted as conferring toxicity on silica (39, 40), recent research suggests that the number and distribution of silanol and siloxane groups rather than crystallinity feature as the primary toxic factors (41).

### Silica and Tuberculosis

Tuberculosis remains one of the most dangerous diseases of the modern world, causing more than 1.3 million deaths, and with over 10 million new (incident) TB cases worldwide, in 2017 (42). Many risk co-factors for TB have been identified, including malnutrition, alcohol, diabetes and drug abuse (43–46). HIV infection is a potent co-factor (42, 47), while a higher incidence of TB has also been observed in individuals suffering from parasitic infections such as malaria (48) and leishmaniasis (49). Inhalant co-factors include smoking (50) and indoor air pollution (51, 52). The co-occurrence of silica exposure, silicosis, and TB has long been identified in populations exposed to silica-containing dust (53, 54) and progression of TB associated with silica exposure or silicosis has been of interest at least since the beginning of twentieth century (55). The spectrum of effect after exposure to the two agents is complex. It ranges from retained silica particles in the lung and TB infection without active disease, to dual disease, referred to as silicotuberculosis. The fibrotic phase of silicosis may be sub-radiological and thus not clinically evident (56), while significant host pathological changes may be observed in the pre-fibrotic stages as a result of silica particle activity (57).

The protection of silica-exposed and silicotic individuals against mycobacterial infection and TB in particular remains an important clinical and public health issue in the twenty-first century (58). Silica dust control, treatment of latent TB infection, early detection and effective treatment of TB are the main modalities of control. However, at the community level, a recent trial of mass treatment of latent TB infection in gold miners failed to show a reduction in TB incidence rates after treatment courses had ended (59). With respect to vaccination, the evidence does not support the use of Bacillus Calmette–Guérin (BCG) in tuberculin negative silica exposed workers or silicotics (60). For example, vaccination with BCG in guinea pigs exposed to silica-containing dust has been reported as causing an increase in fatal BCG infection, while an increased death rate from silicosis and silicotuberculosis has been observed in BCG vaccinated Bulgarian miners (61).

With the objective of understanding exposure-response effects, the risk as well as the progression and severity of mycobacterial infection in the presence of silica exposure have been investigated, using variable dust composition and concentrations of quartz (the most common crystalline silica polymorph), and various inoculation sites of different mycobacterial strains. While tracheal and, in particular, intravenous infection produce extensive pulmonary lesions, a subcutaneous introduction of bacilli did not show any pathological changes. It has also been confirmed that quartz represents the most toxic form and that there is a relationship between increasing dust/silica concentration, number of bacilli and development of tuberculous lesions (62). Co-exposure *in vivo* in guinea pig models has also provided information about the active role of Mtb in pneumoconio-tuberculous lesion formation, with the introduction of the antituberculotic drug rifampicin resulting in decreased formation or its elimination (63).

However, since activation of the immune system underlies disease activation and its accelerated progression, the factors targeting components of protective immune mechanisms and leading to impairment of defense against mycobacterial infections need to be understood. Exposure to silica decreases cellular function, reduces the capacity of dendritic cell activation and leads to a non-specific, impaired inflammatory response which compromises antibacterial mechanisms (64). These effects provide pathways for the promotion by silica exposure of increased susceptibility to bacterial infections, particularly *Mycobacterium tuberculosis* and other mycobacterial species (63, 65, 66).

Detailed mechanisms leading to disease in the presence of silica and Mtb are not yet understood, and multiple pathways are likely to be involved. For example, genetic polymorphism of tumor necrosis factor alpha (TNF-α), natural resistance-associated macrophage protein 1 (NRAMP1), and inducible nitric oxide synthase (iNOS) in macrophages have been shown to influence the response to both silica exposure or silicosis and TB in Chinese iron miners (67). Similar observations have been made in silicotic South African gold miners (68). Based on their specific single nucleotide polymorphism, different genotypes of the above-mentioned proteins showed intricate differential effects. These were either protective (variation of iNOS Ser608Leu genotype in silicosis) or had deleterious effects (G>C mutation of NRAMP1 intron 4 in silicosis, combined NRAMP1 D543N G/G and INT4 G/C+C/C, polymorphic site of G/A substitutions at positions−308 of TNF-α and TNF-a-308 G/G and NRAMP1 INT4 G/C+C/C genotype in TB) (67, 68).

Polymorphism of other genes related to the response to TB and silica such as transforming growth factor-beta 1 (TGF-β1) and cytokines interleukin 10 (IL-10) and interferon gamma (IFNγ) have been investigated, but no association found (69).

The following sections review the innate and adaptive cellular immune responses to silica and Mtb separately and with co-exposure, according to the immune cells or processes involved. The findings are summarized in Table 1, which also highlights the gaps in knowledge.

**Table 1:** Basic summary of acquired knowledge about specific components of the immune system after exposure to silica, Mtb, or both, highlighting the gaps and opportunities for future research.

	**Silica**	**Mtb**	**Silica + Mtb**
Macrophages	Dose-dependent Induction of apoptosis, fibrotic nodule formation, chronic activation of inflammatory and anti-inflammatory pathways, ROS, and RNI production (6)	Granuloma formation, chronic inflammation, survival mechanisms: ROS and RNI detoxification, blockade of phagosome maturation, granuloma formation (70)	Increase in Mtb uptake (71); impairment of adhesion and migration (72, 73)
Dendritic cells	Lower viability (74)	Cells with the highest bacterial burden in lymph nodes (75); increase in antigen presentation and secretion of pro-inflammatory cytokines (76); activation of adaptive immunity (77)	Yet to be investigated
Neutrophils	Decreased phagocytosis and viability (78, 79)	Control of Mtb replication, prolonged activation—tissue damage (80); trapping of bacilli (81); facilitation of disease progression (82)	Yet be investigated
NKs	Decrease in NKs (83, 84); silica nanoparticles cause increase in NKs (85)	Protective role of NKs—production of IFNγ (86)	Yet to be investigated
Antigens	Inconsistent evidence, increase in antigen-presenting properties of AMs (87)	CD1 mediated T cell activation (88, 89); signaling for APCs (90)	Yet to be investigated
T cells CD4+, CD8+, γδ T cells	Increase in FAS ligand—higher rate of apoptosis (91); shift between Th1/Th2 response (92–96); Tregs and Tresps activation (97); reduced inhibitory activity of T cells (98)	Bacteriostatic and bactericidal effect (77); IFNγ production—phagocytic properties of macrophages, Th2 response activation and Tregs production (99, 100)	Sporadic and contradictory evidence
Antibody-mediated immunity (B cells)	Increase and decrease in B cell activity (101); increase in autoantibodies (97)	B cells are the producers of antibodies, modulators of T cell activity and T cell memory, influencing function of dendritic cells (102); association with containment of Mtb (103); CD4+ T cell regulation (104)	Yet to be investigated

## Innate Cellular Immune Responses in Silicosis and Tuberculosis

### Alveolar Macrophages (AMs)

Alveolar macrophages represent the first line of defense against many airborne pathogens and inhaled environmental particles. The reaction of AMs after exposure to silica (70, 105–115) has been extensively studied. The tissue remodeling and formation of granulomas in response to exposure to both silica and Mtb suggest that similar mechanisms are involved in the elimination process in individuals exposed to silica or Mtb separately (116–118). Results from several studies have confirmed a noticeable difference in growth of Mtb in macrophages preloaded with silica particles; macrophages were more susceptible to infection represented by an increase in the number of infected macrophages and a higher number of bacilli present in each cell (119). Thus, silica particles facilitate intracellular replication and subsequent release from macrophages (61).

Using BCG vaccine inoculations, silicotic mice exhibited a higher accumulation of BCG colonies in harvested organs linearly correlating with the length of incubation than naïve counterparts. After 12 weeks, more than 40 times more colonies were observed in the spleen of silicotic mice, 70 times more in the liver and more than 6,000 times more in the lungs. Presence of silica particles significantly affects cellular response and bacterial growth properties (71). Higher Mtb burdens have been observed in murine lungs preloaded with silica compared to silica-free control animals (73). Also, extracted AMs contained more engulfed bacilli. The transplantation of AMs from silica-exposed into control, silica-unexposed mice, resulted in an increase of susceptibility to TB upon infection with Mtb. Thus, macrophages preloaded with silica particles exhibit a higher number of Mtb-phagocyting cells as well as higher rates of Mtb phagocytosis, leading to an increase in the number of bacteria engulfed in the macrophage (120). Silica-containing macrophages also display impaired capability to adhere and migrate compared to healthy controls (72).

Enhanced Mtb phagocytosis could be caused by an interaction with intracellularly located surfactant-associated protein A (SP-A) or its related proteins (121–123) A significantly higher release of SP-A has been shown to follow exposure to silica (124) and this increase was associated with reduced silica-related toxicity to AMs (62, 125) analyzed the impact of various types of dust particles on lung tissue after co-administration with inoculated BCG and specifically on the development of fibrotic lesions. They demonstrated that rats and guinea-pigs developed only mild fibrotic lesions after exposure to mine dust, anthracite, kaolin, and BCG alone but large destructive lesions with combined dust and BCG.

### Dendritic Cells (DCs)

The influence of silica exposure on DCs function has been little investigated. Beamer and Holian (126) describe an increase of DCs accompanying the decrease in numbers of AMs after exposure to silica. It has been reported that viability of DCs is compromised after exposure to silica particles (74). The role of DCs in resistance is also not well-documented in cases of silica potentiated TB. It has been shown that mycobacteria are able to enter the intracellular spaces of DCs via the specific surface protein DC-specific intercellular adhesion molecule-3 grabbing non-integrin (DC-SIGN) (127). Although the main carriers of Mtb are macrophages, the most infected population of cells in lymph nodes are DCs. The evidence is that antigen presentation properties and migration of DCs play important roles in the establishment of long-term infection (75). They also increase their antigen-presenting properties after infection with mycobacteria, facilitate immune system responses through secretion of pro-inflammatory cytokines [tumor necrosis factor alpha (TNFα), IL-1] and up-regulation of co-stimulatory (CD54, CD40) molecules (76). DCs also specifically stimulate the production of IFNγ by T cells after exposure to Mtb (128).

### Neutrophils

Neutrophils rapidly respond to pathogen challenge and contribute to control of Mtb replication. However, apart from its protective properties, the cell's prolonged oxidative and proteolytic activity also lead to tissue damage (80). Neutrophils possess the direct ability to restrict Mtb by engulfment of bacilli (129, 130). Increased influx of neutrophils into Mtb-infected tissue has been observed although in this study no bacteria were found inside these cells. This might point to antibacterial activity independent of phagocytosis (131). Their detailed role in disease is thus not yet fully understood.

It has been found that low concentrations of neutrophils in the peripheral blood cause incapability to restrict or kill introduced Mtb (132). Its oxidative phagocytic properties may kill some of the bacteria after phagocyting the dying macrophage (133). Antimicrobial extracellular traps (NETs) formed by neutrophils are also able to contribute to antimycobacterial activity by trapping bacilli, but not often by killing them (81). Neutrophil-related restriction of mycobacterial growth can also be caused by their role in specific T helper 1 (Th1) and Th17 cell production (134). In contrast, it has been argued that neutrophils possess only poor direct antimycobacterial activity and that they instead facilitate the progression of infection (82). Silica particles are known to harm neutrophils in similar ways to that of their effect on macrophages, by decreasing their viability and phagocytic properties (78, 79).

### Other Cells [Basophils, Eosinophils, Natural Killer (NK) Cells]

Detailed information about other components of the innate immune system in patients with silica-related TB is almost non-existent. A protective role for NK cells has been observed in mouse lung and splenic cultures infected with Mtb. In this study, production of IFNγ by NK cells significantly contributed to limiting mycobacterial infection, independently of IFNγ produced by T cells. Higher mycobacterial burden and granulocytic activity have been observed after depletion of Interleukin 12 (IL-12), which generally promotes NK cell cytotoxic activity (86). A decrease in NK cell number has been observed after the introduction of silica particles (83, 84). Detailed understanding of silica effects on NK cells is still lacking, but it has been observed that silica particles inhibit Toxoplasma-induced NK cell activity (135). By contrast, silica nanoparticles induce a significant increase in NK cells in mouse spleen, which may indicate a size-dependent effect of the particles on NK cells (85).

## Adaptive Cellular Immune Responses in Silicosis and Tuberculosis

### Antigen Presentation

Antigen presentation is generally described as the presentation of lysed proteins on the surface of antigen-presenting cells (APCs) via the Major Histocompatibility Complexes (MHC), which is recognized by T cell receptors leading to their activation. Mycobacterial phospholipids activate CD1-mediated T cells (88, 89), and it has been observed that Mtb provides signaling for both T cells (lipid antigens) and APCs (polar lipids via TLR-2) (90).

Silica, as a pro-inflammatory agent, causes primarily induction of apoptosis of AMs. Various studies indicate that silica does not act as an antigen (136). Rather, silica particles support the increase of antigen-presenting properties of macrophages (87). Enhanced activation of AMs could also be related to autoimmune disease as a result of silica exposure. No significant differences were detected in the expression of MHC II molecules on AMs between silicosis and other diseases, such as sarcoidosis and allergic alveolitis (137). However, Pfau et al. (138) observed that silica-related antigens presented by apoptotic AMs are recognized by autoantibodies in mice. The question of how silica particles modulate presentation of antigen in the relationship with mycobacterial infection remains unanswered.

### T Lymphocytes—CD4+, CD8+, γδ T Cells

**Figure 1:**
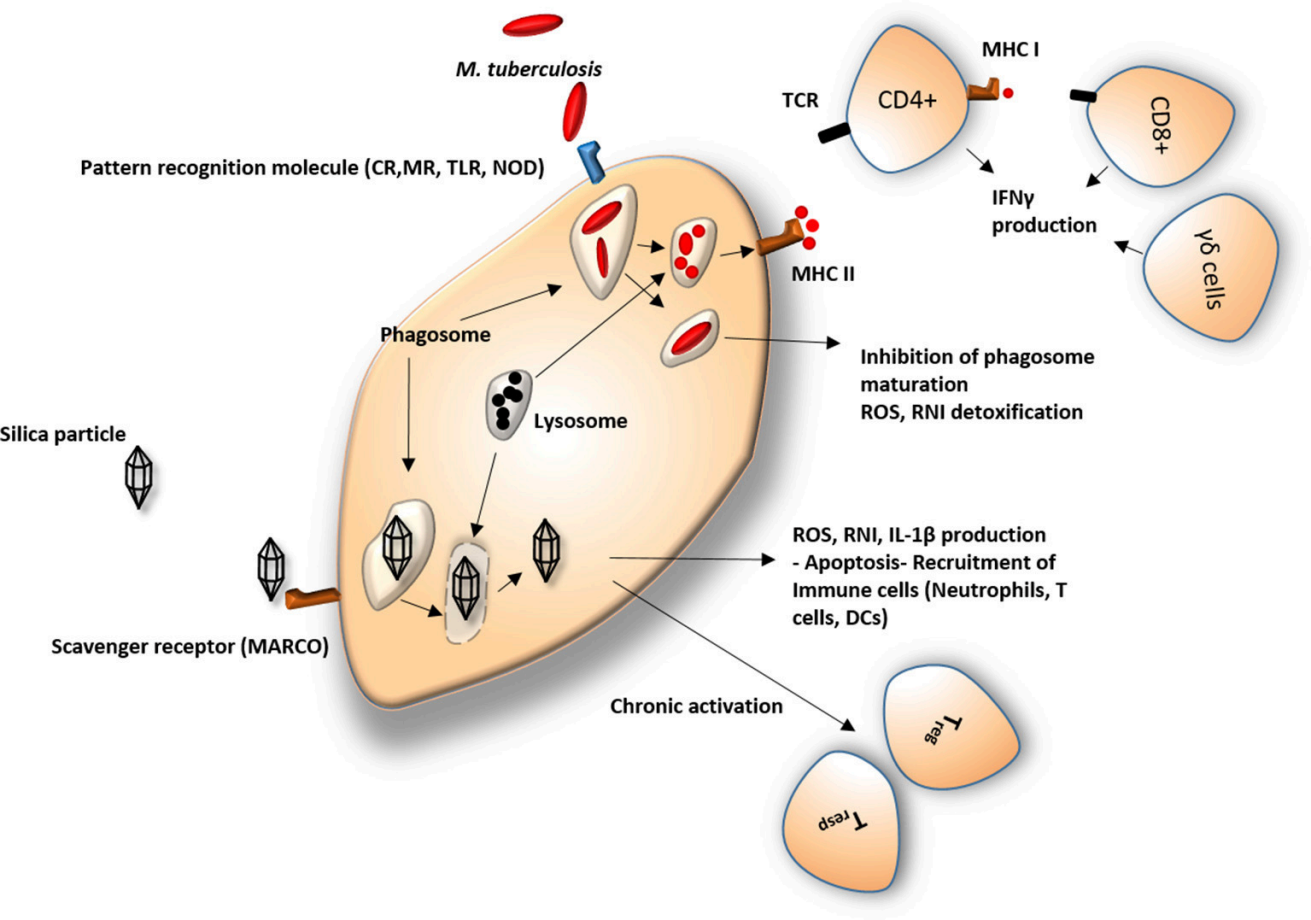
Response of macrophage to silica and *Mycobacterium*. CR, Complement receptors; MR, Mannose receptors; TLR, Toll like receptors; NOD, Nod-like receptors; ROS, Reactive oxygen species; RNI, Reactive nitrogen intermediates; IL-1β, Interleukin 1 beta; MHC I, major histocompatibility complex 1; MHC II, major histocompatibility complex 2; IFNγ, Interferon gamma; TCR, T-cell receptor; DCs, dendritic cells; CD4+/CD8+, effector T cells; T_regs_, regulatory T cells; T_resps_, responder T cells; γδ cells, gamma delta T cells. Silica particles are usually recognized by scavenger receptors and engulfed by macrophages. Owing to the poor capability of the cell in eliminating the particle by standard lysosomal proteolytic enzymes, silica particles remain in the cell and initiate a cascade of pro- and anti-inflammatory processes, leading to a number of pathological modifications in the immune response. Mycobacterium is identified by a different set of cellular receptors but undergoes a similar process upon its ingestion. The physiological response of the immune system consists of digestion of bacilli and its antigen presentation on the cell surface leading to recruitment of effector cells such as CD4+ and CD8+ T cells. Mycobacterial evasive mechanisms frequently manage to avoid elimination, leading to pathological.

T cells activation occurs on exposure to an antigen which is presented by APCs. The major APCs responsible for defense against mycobacterial infection and activators of adaptive immunity are macrophages and dendritic cells. Broad antimycobacterial activity is provided by different subsets of T cells targeting a wide range of specific mycobacterial antigens. It is known, for example, that CD4+, CD8+, γδ T cells, and CD1 restricted T cells response to infection, contributing to bacteriostatic and bactericidal effects (77). CD4+ and CD8+ T cells act as IFNγ producers and facilitate the phagocytic properties of macrophages (Figure 1). The activity of these subsets might be independent of each other, although their combined activity shows the strongest anti-mycobacterial effects (139). The onset of adaptive immunity is initiated with a significant delay after initial exposure to the pathogen. The restrictive activity of T cells is sufficient for bacterial arrest but not sufficient to kill mycobacteria, which might contribute to prolonged incubation and consequent re-activation of disease (140). In case of T cells response, a number of studies have confirmed that mycobacterial infection triggers Th2 response and T regulatory cells (Tregs) production rather than Th1 response. Deficiency in Th1 response may contribute to poor elimination and successful proliferation of Mtb in the host (99, 100).

The response of adaptive immunity components to silica particles is related to the cascade of activities mediated by the interaction with cells involved in innate immunity, particularly macrophages. However, direct interaction of T cells with silica cannot be excluded. It has been observed, that T cells acquire higher expression of FAS receptor and its ligands in lymphocytes obtained from bronchoalveolar lavage (BAL) fluid from silica-exposed individuals. This includes an over 20% increase in Fas receptor and more than 30% in Fas ligand on CD4+, CD8+ CD56+, and CD45RO+ cells, corresponding to the higher rate of apoptosis (30%) (91). Both inflammatory and anti-inflammatory responses have been induced after exposure to silica. However, conflicting results in specific cytokine production have been reported, suggesting that there is a shift between Th1/Th2 responses during disease progression. There is noticeable inconsistency for another cytokine involved in Th1 response, IFN-γ. Its levels in lungs and lymph nodes have been reported as both elevated and undetected in silica-exposed mice. As a result of Th2 response, an increase in production of IL-12 mRNA and IgG_1_ has been reported in silicotic mice and *in vitro* (92, 96).

More recently, attention has been paid to the role of silica in autoimmune responses, in which autoimmune cells are activated by signals from silica-induced apoptotic macrophages (141). Following silica exposure, Tregs and Responder T cells (Tresps) are chronically activated and infiltrate the peripheral T cell population. By increased production of Fas ligand, Treg cells induce apoptosis and impair the immune response (97). Therefore, the overall number and inhibitory activity of T cells are reduced, with Tresps surviving due to an expression of apoptosis inhibitors (98). A closer look at cellular quantitative evaluation shows a silica-induced reduction of a number of macrophages and a consequential decrease of macrophage-dependent activity of T- and NK cells (142).

Some of the features related to the leukocyte response modulation after silica exposure entail inconsistent and contradictory mechanistic effects regarding silica-TB co-occurrence. For example, increased production of TGF-β as a reaction to silica (143) should assist in the process of elimination of Mtb, not its potentiation (144, 145). There is other evidence for a protective effect of silica in its interaction with the immune system, for example in protecting against the development of diabetes (146, 147).

### Antibody-Mediated Immunity

B lymphocytes mediate specific immunological responses against pathogens via the production of specific antibodies. They can modulate responses to infection independent of antibodies; however, the mechanism(s) of such activity is not yet understood (148). The proliferation of plasma cells in patients with silicosis was discovered early (149, 150). Silica particles can cause both an increase and inhibition of B cell activity. Reduced and increased numbers of antibodies have been observed after exposure to silica in animal models. Moseley et al. (101) observed complete inhibition of immunoglobulin-secreting cells (ISCs) after the addition of silica in low- and high-density cultures depleted of monocytes. In contrast, in high-density cultures of unfractionated mononuclear cells, silica caused a significant increase in ISCs. It, therefore, seems that the number of immunoglobulin secreting cells is dependent not just on silica particles but also on other factors such as density of culture and presence of monocytes. A significant reduction in B cells was observed in mouse spleens treated with silica nanoparticles (85). The most common effect of silica was an increase of autoantibodies such as rheumatoid factor (anti IgG), and anti-nuclear antibodies related to autoimmune diseases such as Caplan's syndrome, scleroderma, (ANCA)-related vasculitis/nephritis, and systemic lupus erythematosus (97).

In response to mycobacterial infection, B cells do not simply act as producers of antibodies but also as modulators of T cell activity and development of T cell memory. They also influence the function of other effector cells such as DCs. The importance of B cells during mycobacterial infection is based on the co-operation and co-stimulation with other components of the immune system, suggesting more complex involvement in the immunological response (102). Antibodies in lymphocyte supernatants have been studied to obtain information about its diagnostic potential in pediatric TB patients. The findings indicated that antibodies were present at higher concentrations only during acute disease in TB positive patients compared to controls (151). Proliferating B cells are mostly present in sites of granulomas actively secreting TB-specific antibodies (103, 152) observed that abnormally located B cells in patients with TB are associated with a containment of Mtb and with IL-17 and IL-22 production. The same group also described inhibitory effect of TB-related B cells on Th17 cell activation and therefore its involvement in CD4+ T cell regulation (104). While the overall role of B cells in the immune response to *M. tuberculosis* has been studied extensively (153, 154), its relationship with silica exposure has not yet been documented.

## Conclusion

It is evident that the human immune system plays a central role in pathophysiological processes initiated after silica exposure and which have an impact on the development of TB. A detailed description of silica's involvement in TB infection, course, progression, re-activation and outcome has yet to be properly described. Existing research confirms the substantial role of the innate immune system in both direct defense and in the mobilization of other components of the immune system as a response to TB infection. Such findings are likely to underlie the higher susceptibility of silica-exposed and silicotic individuals to TB. However, there remain a number of gaps in knowledge, as summarized in Table 1.

Recent findings suggest a more complex involvement of adaptive immunity in the containment and elimination of Mtb and its activation in latent infection. The effects of the immune response to silica exposure are complex and do not always lead to increased susceptibility to TB; some promote the elimination of mycobacteria rather than their proliferation. For example, silica-induced Th1 response activation, mediating TNFα and IFNγ production, should in theory contribute to resistance to or elimination of Mtb. An increase in Th2 response after exposure to silica might be one of the contributing factors for successful propagation of Mtb. Discrepancies in evidence of pro- and anti-inflammatory pathway involvement, however, suggest more intricate reactions promoting fibrogenesis which might make a direct contribution to predisposition to TB.

The subject of this review has clinical relevance. The pathological activity of silica is important in the susceptibility to and prognosis of associated TB in silica-exposed populations globally. The considerations in this review should inform studies that aim to investigate outcomes of standard TB treatment regimens as they apply to silicotuberculosis by providing insight into mechanisms relevant to drug efficacy.

Finally, knowledge gained from the study of the silica-TB interaction could provide information relevant to the understanding of other pathological processes associated with silica exposure.

## Author Contributions

The subject of the review was co-conceived by all the authors. PK conducted the literature search and drafted the manuscript. RE, MG, and MJ contributed substantial intellectual content to the contents of the review and its finalization. All authors take responsibility for the final manuscript.

### Conflict of Interest Statement

The authors declare that the research was conducted in the absence of any commercial or financial relationships that could be construed as a potential conflict of interest.
